# Asynchronous development of bilateral tubal pregnancy after IVF-ET: A rare case report and review of the literature

**DOI:** 10.1097/MD.0000000000043619

**Published:** 2025-08-08

**Authors:** Deying Ban, Qian Deng, Dazun Shi

**Affiliations:** a Clinical Research Center for Reproduction and Genetics in Hunan Province, Reproductive & Genetic Hospital of CITIC-Xiangya, Changsha, Hunan Province, China; b NHC Key Laboratory of Human Stem Cell and Reproductive Engineering, Xiangya School of Basic Medical Sciences, Central South University, Changsha, Hunan Province, China; c Department of Gynecology, Xiangya Hospital, Central South University, Changsha, Hunan Province, China; d Department of Plastic Surgery, The Second Affiliated Hospital of Zhejiang University College of Medicine, Hangzhou, Zhejiang Province, China; e Hunan Engineering Research Center of Skin Health and Disease, Xiangya Hospital, Central South University, Changsha, Hunan Province, China.

**Keywords:** Bilateral Tubal Pregnancy, Asynchronous Development, IVF-ET, Laparoscopic Salpingectomy, Case Report

## Abstract

**Rationale:**

Ectopic pregnancy (EP) is defined as the implantation and development of blastocysts outside the uterine cavity. Unilateral tubal pregnancy accounts for approximately 90% of all EP cases, whereas bilateral tubal pregnancy (BTP) is a rare variant. The incidence of BTP ranges from 1 in 750 to 1 in 1,580 of all ectopic pregnancies and is more common in women who have undergone assisted reproductive technology (ART) than in those with natural conception. However, asynchronous development in the BTP is even rarer.

**Patient concerns:**

A 32-year-old woman underwent embryo transfer at our institution due to primary infertility (In vitro fertilization and embryo transfer with two Day 3, grade I, 8-celled fresh embryos). Nineteen days after the transfer, the patient experienced lower abdominal pain and vaginal bleeding. The β-HCG blood test revealed an elevated level of 7232 IU/L, and ultrasound imaging suggested right tubal pregnancy. Consequently, laparoscopic surgery was performed. Intraoperatively, the isthmus of the right fallopian tube appeared swollen, whereas the left fallopian tube appeared normal. Right salpingectomy was performed, and pathological examination confirmed the diagnosis of right tubal pregnancy. Twelve days postoperatively, the patient exhibited an asymptomatic elevation in β-HCG levels, and ultrasound imaging suggested left tubal pregnancy. The second laparoscopic examination revealed a thickened isthmus in the left fallopian tube.

**Diagnoses:**

Bilateral tubal pregnancy with asynchronous development.

**Interventions:**

Laparoscopic right salpingectomy and left salpingotomy was performed.

**Outcomes:**

Two weeks post-surgery, during follow-up, the patient reported no discomfort, and serum HCG levels normalized.

**Lessons:**

Although asynchronous development of bilateral tubal pregnancy is an extremely rare form of ectopic pregnancy, women who have undergone ≥2 embryos transfer or ovulation induction treatment should be vigilant for the possibility of the above situation.

## 1. Introduction

Ectopic pregnancy (EP) is defined as the implantation and development of the blastocyst outside the uterine cavity. The leading risk factors for EP in women are pelvic inflammatory disease, previous pelvic surgery, tubal infertility, and congenital uterine abnormalities.^[1]^ Approximately 1% to 2% of all pregnancies are EPs, and over 90% of implantations occur in the fallopian tube, while bilateral tubal pregnancy (BTP) is considered a very rare variant.^[2]^ The incidence of BTP accounts for approximately 1/750 to 1/1580 of all EPs,^[3,4]^ it is difficult to estimate the accurate frequency based on case reports. BTP has a higher incidence in women undergoing assisted reproductive technology than in those undergoing natural pregnancy,^[5]^ which may be associated with ovulation induction treatment or multiple embryo transfer. Notably, the asynchronous development of the BTP is even rarer.

## 2. Case presentation

On November 11, 2021, 22 days after embryo transfer, a 32-year-old female patient was admitted to the Department of Gynecology at Xiangya Hospital, Central South University, presenting with abdominal pain for 20 days and vaginal bleeding for 4 days. The patient had a history of primary infertility and polycystic ovary syndrome for 5 years, characterized by an irregular menstrual cycle ranging from 30 to 60 days, with a 6-day duration. B-ultrasound revealed polycystic changes in both ovaries. Hysterosalpingography conducted at another hospital in 2020 indicated that the left fallopian tube was patent, whereas the right fallopian tube was obstructed. On the third day of menstruation, serum levels of follicle-stimulating hormone, luteinizing hormone, estradiol, testosterone, and progesterone were 6.4 mIU/mL, 18.7 mIU/mL, 33.4 pg/mL, 0.21 ng/mL, and 0.1 ng/mL, respectively.

Following 2 unsuccessful cycles of artificial insemination, the patient underwent in vitro fertilization and embryo transfer (IVF-ET) at our Reproductive Medicine Center starting on October 6, 2021 (day 1 of menstruation cycle). After ovarian stimulation, oocyte retrieval, and embryo culture, the patient couple obtained 7 cleavage-stage embryos. Three days after oocyte retrieval (day 17 of the menstrual cycle), her endometrial thickness was measured at 9.6 mm, after which she underwent ultrasound-guided embryo transfer with 2 fresh day 3 cleavage-stage embryos (both grade I, 8-cell embryos).On the 13th day of embryo transfer, the patient requested to check the human chorionic gonadotropin (HCG) in advance. The result was 187 mIu/mL, confirming early pregnancy.

**Figure 1.**
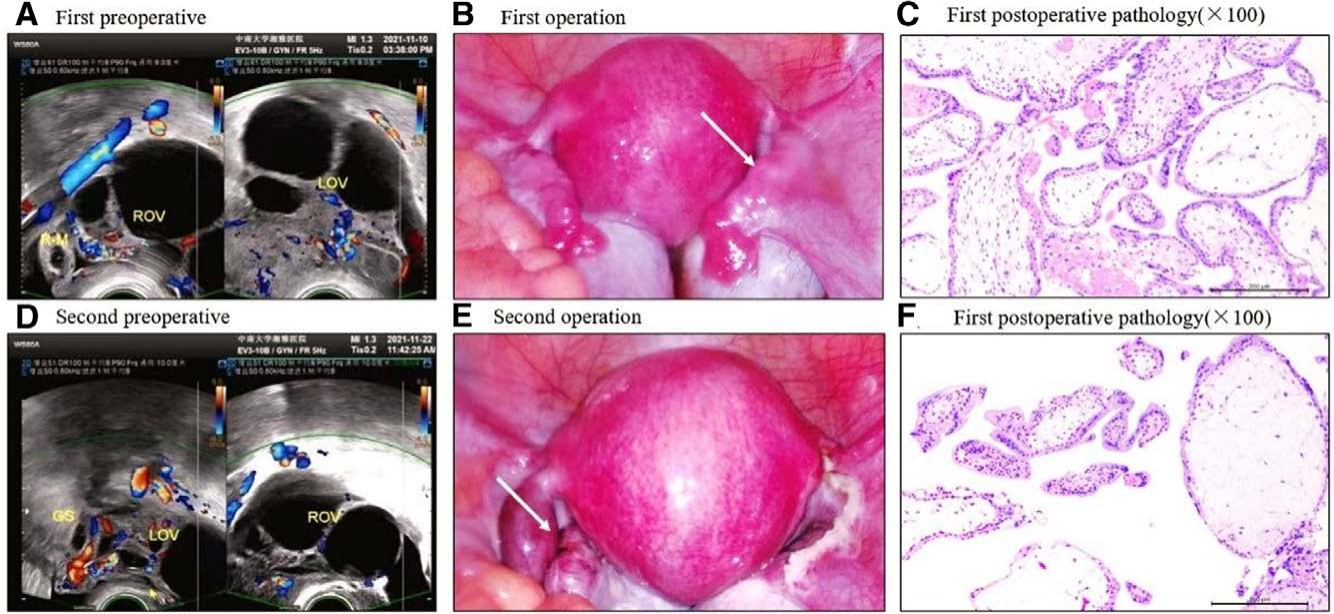
Preoperative ultrasound, surgical images, and postoperative pathology from both procedures (A–F). (A) There is a mixed echo mass next to the right adnexal (size: 19 × 16 × 17 mm) and a gestational sac can be seen in the middle (size: 9 × 8 × 10 mm), in which the inner diameter of the yolk sac is about 3 mm. (B) The isthmus of the right fallopian tube was slightly enlarged and the ovaries on both sides were enlarged (white arrows). (C) Pathological results showed that it was gestational tissue. (D) There is a gestational sac-like echo next to the left ovary (size: 13 × 12 × 10 mm), in which an embryo with a length of approximately 2 mm can be seen. (E) The isthmus of the left fallopian tube and ovaries on both sides were enlarged (white arrows). (F) Pathological results showed that it was gestational tissue. GS = gestational sac, LOV = left ovary, ROV = right ovary.

On the 19th day of embryo transfer, the patient experienced abdominal pain accompanied by minimal vaginal bleeding. Serum β-HCG level was 7232 IU/L. Transvaginal ultrasonography revealed a mixed echogenic mass adjacent to the right ovary (19 × 16 × 17 mm), suggestive of right tubal pregnancy (Fig. 1A). Subsequent laparoscopic exploration confirmed the initial diagnosis with no apparent abnormalities in the left fallopian tube (Fig. 1B). A laparoscopic right salpingectomy was performed. Pathological examination confirmed the presence of villi and proliferative trophoblast cells, thus validating the diagnosis (Fig. 1C).

**Figure 2.**
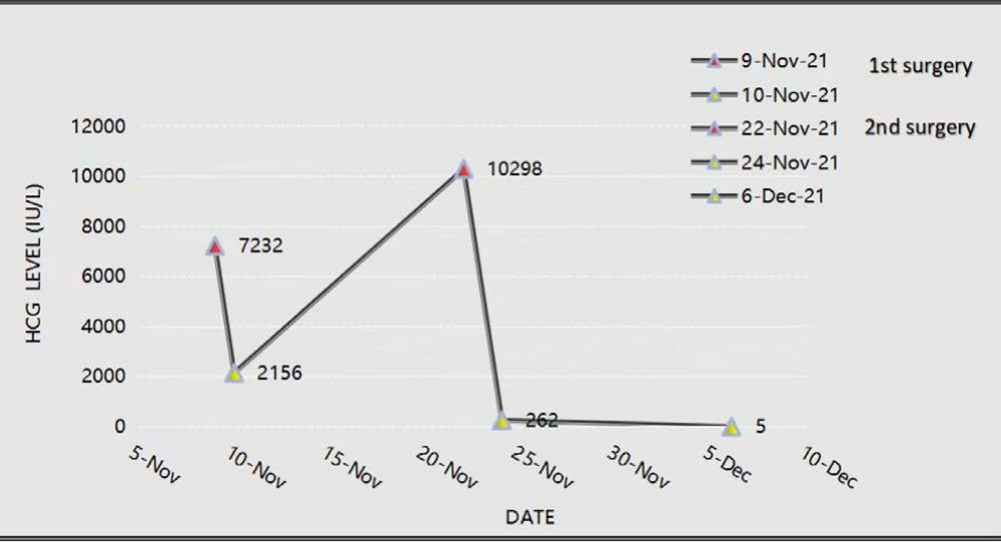
HCG variation diagram. HCG = human chorionic gonadotropin.

Postoperatively, serum β-HCG levels decreased to 2156 IU/L but rebounded to 10,298 IU/L during routine reexamination 12 days later in the outpatient department. Transvaginal ultrasound identified revealed a gestational sac-like echo adjacent to the left ovary (13 × 12 × 10 mm) subsequently (Fig. 1D). A second laparoscopic exploration confirmed left isthmus tubal pregnancy (Fig. 1E), leading to laparoscopic left salpingotomy and embryo extraction. Pathological examination confirmed the presence of villi and trophoblasts (Fig. 1F). Two weeks postsurgery, during follow-up, the patient reported no discomfort, and serum HCG levels normalized (Fig.2).

## 3. Discussion

Considering the high HCG level and the obvious concerns shown by the patient, both the patient and the doctor chose surgical treatment for left tubal pregnancy as the preferred option. Since one fallopian tube had been removed, and although there were still frozen embryos, the patient still hoped to preserve one fallopian tube in the hope of a natural pregnancy in the future. Therefore, the patient eventually underwent laparoscopic left fallopian tube ostomy to remove the pregnancy tissue.

Looking back at this case, if the patient’s symptoms are mild (such as abdominal pain, rapid enlargement of the fallopian tube mass), and they are willing to undergo a relatively longer treatment period, the conservative treatment method of intramuscular injection of methotrexate can still be considered to reduce surgery-related complications and risks.^[6]^

Most bilateral tubal pregnancies develop simultaneously^[7]^; however, asynchronous bilateral tubal pregnancy (ABTP) is exceedingly rare. To gather the most current information on this topic, we conducted a comprehensive search of the MEDLINE, Pubmed and CNKI (China National Knowledge Infrastructure) databases for case reports published since 1980, Searching for literature published in English and Chinese, and using the keywords “bilateral ectopic pregnancy” and “bilateral tubal pregnancy,” published in English and Chinese. Our search yielded only 11 reported ABTP cases (Table 1). Of these, 7 of 11 (64%) cases occurred after infertility treatment, confirming that BTP is commonly associated with infertility treatment.^[5]^ Multiple ovulations, either spontaneous or induced by ovulation induction, seem to elevate the risk of bilateral EP.^[8–10]^

**Table 1:** Summary of the 11 cases reported of asynchronous development of bilateral tubal pregnancy in the medical literature.

Cases	Author	Year	Conception	1st site of ectopic pregnancy	2nd site of ectopic pregnancy	Interval time	Intervention
1	Jean-noël Hugues et al^[14]^	1995	IVF-ET	Right fallopian tube	Left fallopian tube	3 wk	Bilateral salpingectomy
2	Tabachnikoffet al^[13]^	1998	—	Right fallopian tube	Left fallopian tube	7 wk	Bilateral salpingectomy
3	Ryan and Saldana^[15]^	2000	Spontaneous	Left fallopian tube	Right fallopian tube	23 d	Bilateral salpingectomy
4	Khong and Dimitry^[12]^	2005	OI (clomiphene)	Left fallopian tube	Right fallopian tube	1 mo	Bilateral salpingectomy
5	Gong and Tu^[11]^	2012	IVF-ET	Right fallopian tube	Left fallopian tube	7 d	Right salpingectomy + MTX
6	Zhu et al^[9]^	2014	OI	Right fallopian tube	Left fallopian tube	22 d	Bilateral salpingectomy + MTX
7	Gardyszewska et al^[10]^	2016	spontaneous	Left fallopian tube	Right fallopian tube	21 d	Left salpingostomy + laparoscopic surgery
8	Gerli et al^[16]^	2016	OI (clomiphene)	Right fallopian tube	Left fallopian tube	20 d	Right salpingectomy + left salpingostomy
9	Zhou et al^[17]^	2017	Spontaneous	Right fallopian tube	Left fallopian tube	12 d	Right salpingostomy + left salpingectomy
10	Trindade et al^[8]^	2019	IVF-ET (donor eggs)	Left ovary	Right fallopian tube	1 wk	Excision of ectopic tissue + right salpingectomy
11	Mansouri et al^[18]^	2023	OI (letrozole)	Left fallopian tube	Right adnexal	2 wk	Left salpingostomy + MTX

IVF-ET = in vitro fertilization and embryo transfer, MTX = methotrexate, OI = ovulation induction.

The literature indicates that the diagnosis of a second EP in cases of BTP can be delayed by several days to up to 7 weeks following the initial surgical intervention.^[8,11–18]^ The earliest report retrieved was published by Hugues et al^[14]^ in 1995, which described a woman who underwent IVF-ET and was diagnosed with asynchronous bilateral EP after 3 weeks. However, this report lacked image data. In 2019, Brazilian researchers documented a case in which a patient developed unilateral ovarian pregnancy 25 days after embryo transfer and was subsequently diagnosed with contralateral tubal pregnancy 8 days later.^[8]^ Early stage β-hCG levels are not specific enough to diagnose unilateral or bilateral EP, and typical clinical signs such as abdominal pain and vaginal bleeding are nonspecific. Therefore, when 1 EP is confirmed, it is easy to overlook delayed EPs that develop asynchronously.

By retrospectively comparing intraoperative and ultrasound images from our hospital (Fig. 1), it is evident that embryonic development in the left fallopian tube significantly lagged behind that in the right fallopian tube. During the routine intraoperative examination, no abnormalities were observed in the appearance of the left fallopian tube during the first operation. According to the medical history, there was no sexual activity between the couple during IVF-ET preparation, thus excluding the possibility of natural conception. Therefore, we believe that after the transfer of the same grade of 2 embryos, the development of the embryo that mistakenly landed in the left fallopian tube lagged behind that in the right by approximately 12 to 14 days, suggesting an extremely rare asynchronous development of the BTP.

To date, the mechanism underlying ABTP has not been clearly elucidated. From the only available literature and patient presentations, we can infer that this rare phenomenon can be attributed to 3 interrelated dimensions: Time factors, hormonal environmental factors, and spatial factors. Each factor may operate through different mechanisms, but their interaction ultimately shapes the behavior of the system.

### 3.1. Time factors

Operations, such as promoting ovulation or transferring 2 or more embryos, may lead to different degrees of embryonic development or inconsistent implantation times. This phenomenon, known as “diapause” or “germ cell dormancy,” has been observed in insects, copepods, and over 130 species of mammals, including giant pandas and kangaroos.^[19,20]^ Bulut-Karslioglu et al found that mouse blastocysts can enter a dormant state by inhibiting key factors in the mammalian target of rapamycin pathway, yet dormant blastocysts retain pluripotency and the ability to produce embryonic stem cells and viable mice.^[21]^ Given that the transfer window for embryo implantation is the optimal period for endometrial receptivity, embryonic dormancy may be related to decreased uterine receptivity and imbalanced local estrogen and/or progesterone levels. Therefore, we speculated that human embryos might experience dormancy during implantation into the endometrium. Although measuring the time from fertilization to implantation in humans is challenging and current research has not definitively identified diapause in human embryos, the occasional long intervals between artificial embryo transfer and confirmed pregnancy in vitro fertilization suggest that human embryos may also undergo diapause in certain cases, potentially explaining the asynchronous development observed in this case.

### 3.2. Hormonal environmental factors

Progesterone drugs, such as progesterone and dydrogesterone, are commonly used postembryo transfers to provide luteal support and reduce uterine contraction and tubal peristalsis. However, studies have shown that in the nonhuman primate, cyclic changes in circulating progesterone against a background of estradiol stimulate changes in oviductal ciliary beating, muscular contraction, and oviductal fluid volume and composition.^[22]^ Progesterone can both suppress proinflammatory and stimulate anti-inflammatory responses in the oviduct.^[23]^ Therefore, it can be concluded that a certain threshold of progesterone level may cause the balance of inflammatory factors in the fallopian tube to be broken, and affect the frequency of tubal cilia oscillation, thereby promoting EP, or even asynchronous bilateral EP.

### 3.3. Spatial factors

The patient developed mild ovarian hyperstimulation syndrome following fresh embryo implantation. Significantly enlarged bilateral ovaries may alter the normal anatomical position of the fallopian tubes, thereby increasing the risk of bilateral EP.^[24,25]^

## 4. Conclusion

The ABTP is a rare complication that can occur when assisted reproductive technology is used to assist pregnancy. Physicians involved in reproductive medicine should have a comprehensive understanding of the ABTP. First, the number of implanted embryos should be strictly controlled to minimize risks. Second, for women undergoing multiple embryo transfers, clinicians should be vigilant of the possibility of asynchronous bilateral EPs. Even when diagnosing unilateral EP, it is crucial to conduct routine bilateral exploration during surgery and perform dynamic monitoring of postoperative β-HCG levels. Further interdisciplinary studies are necessary to explore the mechanisms underlying human embryonic diapause. Strong evidence confirming this phenomenon in human embryos could have significant implications in reproductive medicine, oncology, regenerative medicine, and other fields.

## Author contributions

**Data curation:** Deying Ban, Qian Deng.

**Resources:** Deying Ban, Qian Deng, Dazun Shi.

**Writing—original draft:** Deying Ban, Qian Deng.

**Writing—review & editing:** Deying Ban, Dazun Shi.

**Conceptualization:** Dazun Shi.

**Project administration:** Dazun Shi.

**Supervision:** Dazun Shi.
